# Transcriptome analysis of *Ginkgo biloba* kernels

**DOI:** 10.3389/fpls.2015.00819

**Published:** 2015-10-06

**Authors:** Bing He, Yincong Gu, Meng Xu, Jianwen Wang, Fuliang Cao, Li-an Xu

**Affiliations:** Co-Innovation Center for Sustainable Forestry in Southern China, Nanjing Forestry UniversityNanjing, China

**Keywords:** *Ginkgo biloba*, kernel, differentially expressed genes (DEGs), terpenoid backbone biosynthesis, transcriptome

## Abstract

*Ginkgo biloba* is a dioecious species native to China with medicinally and phylogenetically important characteristics; however, genomic resources for this species are limited. In this study, we performed the first transcriptome sequencing for *Ginkgo* kernels at five time points using Illumina paired-end sequencing. Approximately 25.08-Gb clean reads were obtained, and 68,547 unigenes with an average length of 870 bp were generated by de novo assembly. Of these unigenes, 29,987 (43.74%) were annotated in publicly available plant protein database. A total of 3,869 genes were identified as significantly differentially expressed, and enrichment analysis was conducted at different time points. Furthermore, metabolic pathway analysis revealed that 66 unigenes were responsible for terpenoid backbone biosynthesis, with up to 12 up-regulated unigenes involved in the biosynthesis of ginkgolide and bilobalide. Differential gene expression analysis together with real-time PCR experiments indicated that the synthesis of bilobalide may have interfered with the ginkgolide synthesis process in the kernel. These data can remarkably expand the existing transcriptome resources of *Ginkgo*, and provide a valuable platform to reveal more on developmental and metabolic mechanisms of this species.

## Introduction

*Ginkgo biloba* L. is a long-lived dioecious plant. The species is endemic in China and is the only known member of the Ginkgopsida. Modern classification based on phylogeny recognizes the species in a separate order Ginkgoales. All living gymnosperms were divided into four orders, Cycadales, Gingoales, Gnetales, and Pinales/Coniferales. As one of the most ancient seed plants, the earliest *Ginkgo*-like trees can be traced back to the early Permian period, approximately 280 million years ago(Zhou and Zheng, 2003; Gong, 2008). *Ginkgo* trees flourished in the Jurassic and Cretaceous periods of the Mesozoic era, playing important roles in the biosphere. Since its unique morphology and physiology characteristics, together with many debates on recent phylogenetic studies of seed plants (Wu et al., 2013), they played important roles in the research of evolutionary relationships.

Apart from its special evolutionary status, *Ginkgo biloba* also possesses considerable medical value. *Ginkgo biloba* extract (GBE) is among the best-selling herbal remedies in America and many European countries (Kressmann et al., 2002; Yilmaz et al., 2010). It has been used to treat cardiovascular and cerebrovascular diseases (Lin et al., 2002). A standardized preparation of GBE, EGB761, is used to treat thrombosis, inflammation, cardiovascular conditions, and Alzheimer’s disease (Oken et al., 1998; Sasaki et al., 2002; Rodríguez et al., 2007). The components of GBE are very complex, and its major medical ingredients are flavonoids and terpenoids (Horáková et al., 2003; van Beek, 2005). These terpenoids primarily consist of ginkgolide and bilobalide, both of which are rare terpene trilactone compounds.

As the case for all other isoprenoid compounds, the syntheses of ginkgolide and bilobalide involve three similar steps: (1) the formation of isopentenyl diphosphate (IPP) and its isomer dimethylallyl diphosphate (DMAPP); (2) the repetitive condensation of IPP and DMAPP to their precursors farnesyl diphosphate (FPP) and geranylgeranyl diphosphate (GGPP); and (3) the late cyclization and oxidation of these compounds as catalyzed by terpenoid synthesis and cytochrome P450 -dependent monooxygenases. After these common steps, ginkgolide and bilobalide are synthesized through the methylerythritol 4-phosphate (MEP) and mevalonic acid (MVA) pathways, respectively (Rohmer, 1999; Kim et al., 2006b). It is suggested that bilobalide is a sesquiterpene which contains three lactones units (Sabater-Jara et al., 2013), and the specific biosynthetic mechanism of bilobalide in *G. biloba* remains unclear, although many genes coding for key enzymes involved in the MEP pathway have been cloned. The gene coding for 3-hydroxy-3-methylglutaryl coenzyme A (*HMG-CoA*), which is related to the first committed step of bilobalide synthesis, has been cloned and identified (Shen et al., 2006).

Though ginkgolides are present in both leaves and roots, the sites of terpene trilactone biosynthesis remain unclear. Recently, several functional genomics studies have been conducted on *G. biloba* (Wang et al., 2010; Lin et al., 2011, 2012), and these previous studies have mostly focused on leaves rather than kernels. However, the ginkgo leaves contain only small amounts of ginkgolides (Nakanishi, 1967) and people have been eating *G. biloba* kernels directly for 1000s of years, believing it beneficial for health. According to many researches, genes coding for enzymes of the MEP pathway involved in IPP and DMAPP are highly expressed predominantly in the roots (Gao et al., 2006; Kim et al., 2006a,b; Shen et al., 2006). Ginkgolides would be synthesized in the roots and then transported to the stems and leaves (Kim et al., 2012). As a sesquiterpene, detailed evidence of bilobalide formation is still lacking. In this study, we attempted to reveal the synthesis or regulative mechanism of ginkgolide and bilobalide in *Ginkgo* kernels by Illumina paired-end sequencing and bioinformatics analysis and we identified several useful differentially expressed genes (DEGs) involved in both pathways.

## Materials and Methods

### Plant Materials

Seeds from a mature *Ginkgo* tree growing at Nanjing Forestry University were collected at different developmental time points in 2013 (8 July: Gb_Seed1; 5 August: Gb_Seed2; 2 September: Gb_Seed3; 20 November: Gb_Seed4; 2 December: Gb_Seed5), after the removal of the testae and then quickly frozen in liquid nitrogen and stored at -80°C until RNA extraction was conducted.

### RNA Extraction and Library Preparation

Total RNA extraction was performed according to the manufacturer’s instructions using the RNeasy Plant Mini Kit (Qiagen, Hilde, Germany). Before extraction, RNA degradation and contamination were monitored on 1% agarose gels. RNA purity was checked using a NanoPhotometer^®^spectrophotometer (Implen, Westlake Village, CA, USA). RNA concentration was measured using the Qubit^®^RNA Assay Kit in a Qubit^®^2.0 Flurometer (Life Technologies, Carlsbad, CA, USA). RNA integrity was assessed using the RNA Nano 6000 Assay Kit in an Agilent Bioanalyzer 2100 system (Agilent Technologies, Santa Clara, CA, USA). A total of 3 μg RNA was used as an input per sample. Sequencing libraries were generated using a NEBNext^®^Ultra^TM^ RNA Library Prep Kit for Illumina^®^(NEB, USA) following the manufacturer’s instructions, and index codes were added to attribute sequences to each sample. Clustering of the index-coded samples was performed on a cBot Cluster Generation System using the TruSeq PE Cluster Kit v3-cBot-HS (Illumia) according to the manufacturer’s instructions. After cluster generation, the library preparations were sequenced on an Illumina Hiseq 2000 platform, and paired-end reads were generated.

### Data Processing, Assembly, and Annotation

Raw data (raw reads) in FASTQ format were first processed using in-house Perl scripts. In this step, clean data (clean reads) were obtained by removing adapter-containing, poly-N, and low-quality reads from the raw data. At the same time, the Q20, Q30, GC content, and sequence duplication level of the clean data were calculated. All downstream analyses were based on high-quality, clean data. The left files (read1 files) from all libraries/samples were pooled into a single left.fq file, and the right files (read2 files) were pooled into a single right.fq file. Transcriptome assembly was accomplished from the left.fq and right.fq using Trinity (Grabherr et al., 2011), with min_kmer_cov set to 2 by default and all other parameters set as their defaults. The transcripts of each gene with the longest length were selected as unigenes.

All assembled unigenes were searched against the Nr (NCBI non-redundant protein sequences) database using the BLAST algorithm to study the functions of mRNA and identified homologues of genes with known functions. The best gene ontology (GO) terms acquired were searched against the Nr database using Blast2GO and self-written programs (Götz et al., 2008). The assembled unigenes were also searched against the NT (NCBI nucleotide sequences), Pfam (Protein family), KOG (euKaryotic Ortholog Groups)/COG (Clusters of Orthologous Groups of proteins), Swiss-Prot (a manually annotated and reviewed protein sequence database), and KO [KEGG (Kyoto Encyclopedia of Genes and Genomes) ORTHOLOG] databases to find and predict functional classifications and molecular pathways.

### Differential Expression Analysis

The transcriptome based on the Trinity assembly was set as the reference sequence (ref) for the analysis of gene expression levels. The clean reads of each sample were mapped to the ref, and the number of read counts mapped to each gene was calculated. For this process, we used RSEM software (Li and Dewey, 2011), and the bowtie parameter was set at mismatch 2. The read counts were then transformed with the FPKM method (expected number of fragments per kilobase of transcript sequence per millions of base pairs sequenced), and the impacts of sequencing depth and gene length on fragment counts were also considered (Trapnell et al., 2010). The input data of the DEGs were based on the read counts. In this study, we selected expressed genes of GB_Seed1 as the reference and all the DEGs were generated based on the comparison with it.

Statistical treatment was first performed on the read counts using the trimmed mean of *M*-values method, then differential expression in the digital gene expression data was analyzed using a model based on the negative binomial distribution with the DEGseq (2010) R package. The *p*-value was adjusted using the *q*-value (Storey and Tibshirani, 2003). A *q*-value <0.005 and a log2 fold change >1 were set as the thresholds for significantly differential expression (Anders and Huber, 2010). The identified DEGs were clustered using k-means method and then were used for further GO and KO enrichment analysis. GO enrichment analysis of the DEGs was implemented by the GOseq R packages, based on the Wallenius non-central hypergeometric distribution (Young et al., 2010), which can adjust for gene length bias. We used KOBAS (Mao et al., 2005) software to test the statistical enrichment of DEGs in the KEGG pathways.

### Real-time Quantitative PCR Analyses

Three genes involving in the terpene lactones synthesis pathway (Gene ID: comp27939_c0, comp36057_c0, comp56812_c0) were analyzed using qRT-PCR. New plant materials from the individuals were used for the RNA extraction for the qRT-PCR, and three biological replicates were made. Gene-specific primers were designed according to the reference unigene sequences using online primer-design software (http://www.genscript.com.cn/technology-support/on-line-tools). A HiScriptTM Q RT SuperMix for qRT-PCR (Vazyme, Nanjing, China) was used to synthesize the cDNAs and real-time quantification was performed using a ABI Viia7 Real-Time PCR system and the EvaGreen 2X qPCR Master Mix (Vazyme, Nanjing, China). The PCR cycling was denatured using a program of 95°C for 10 min, and 40 cycles of 95°C for 15 s and 60°C for 60 s. The18S gene in *G. biloba* was selected as the reference gene.

## Results

### Sequencing and Assembly

Illumina sequencing data from *G. biloba* kernels at different time points were deposited in the NCBI SRA database under accession number SRP062414. The results of the high-throughput sequencing were designated as raw data or raw reads, which were stored in FASTQ format (fq) and contained sequence and quality information. In this study, a total of 25.08 Gb of sequencing data were generated, comprising 185,075,486 raw reads and 167,215,904 clean reads. The base average error rate was 0.08%, and the average Q20 and Q30 values were 95.24 and 87.12%, respectively. The average GC content was 45.50% (**Table 1**).

**Table 1:** Summary of sequencing quality.

Sample	Raw reads	Clean reads	Clean bases	Error (%)	Q20 (%)	Q30 (%)	GC (%)
GB_Seed1_1	18,444,811	16,726,624	2.51G	0.07	96.69	89.11	47.76
GB_Seed1_2	18,444,811	16,726,624	2.51G	0.11	93.21	83.38	47.78
GB_Seed2_1	17,635,444	15,887,677	2.38G	0.07	96.74	89.54	45.67
GB_Seed2_2	17,635,444	15,887,677	2.38G	0.11	93.55	84.34	45.68
GB_Seed3_1	18,608,369	16,698,946	2.5G	0.07	96.55	89.11	45.10
GB_Seed3_2	18,608,369	16,698,946	2.5G	0.1	93.64	84.49	45.13
GB_Seed4_1	17,072,541	15,448,434	2.32G	0.07	96.68	89.52	44.43
GB_Seed4_2	17,072,541	15,448,434	2.32G	0.09	94.42	86.25	44.46
GB_Seed5_1	20,776,578	18,846,271	2.83G	0.07	96.72	89.64	44.46
GB_Seed5_2	20,776,578	18,846,271	2.83G	0.09	94.22	85.83	44.48
Summary	18,5075,486	16,721,5904	25.08G				

*(1) Reads sequencing from the left; (2) Reads sequencing from the right.*

*Q20: percentage of bases with a Phred value >20; Q30: percentage of bases with a Phred value >30.*

A total of 112,946 transcripts were generated; the shortest was 201 bp, and the longest was 18,087 bp. The average length was 1262 bp, and the N50 was 2269 bp. A total of 68,547 unigenes were assembled, for which the shortest and longest lengths were the same as those of the transcripts. Among the unigenes, 38,932 (56.80%) were 200–500 bp, 11,658 (17.01%) were 0.5–1 kbp, 9893 (14.43) were 1–2 kb, and the remaining 8064 (11.76%) were >2 kb (**Table 2**).

**Table 2:** Length distribution of assembled transcripts and unigenes.

Nucleotide length	Transcripts	Unigenes
200–500 bp	46,171	38,932
0.5–1 kbp	19,205	11,658
1–2 kbp	23,080	9,893
>2 kbp	24,490	8,064
Total	112,946	68,547
Min length (bp)	201	201
Mean length (bp)	1,262	870
Max length (bp)	18,087	18,087
N50 (bp)	2,269	1,693

### Functional Annotation and Classification

All of the 68,547 assembled unigenes were searched against the Nr, NT, KO, KOG/COG, and Swiss-Prot databases using the BLAST algorithm (*E*-value < 1E-5) and annotated accordingly (Supplementary File S1). Of these unigenes, 26,334 had significant similarity to entries in the Nr database, accounting for 38.41% of the total. A total of 20,331 (29.65%) unigenes were annotated as unknown. The numbers of sequences that were highly similar to entries in the NT, KO, PFAM, GO, and KOG/COG databases were 9721 (14.18%), 5164 (7.53%), 20,197 (29.46%), 21,925 (31.98%), and 10,268 (14.97%), respectively, and 43.74% of the unigenes were annotated in at least one database (**Table 3**). Of unigenes that were not annotated in any of the databases, more than 1,000 unigenes didn’t have open reading frames.

**Table 3:** Summary for the annotations of *Ginkgo biloba* unigenes.

	Number of unigenes	Percentage (%)	Functional categories
Annotated in NR	26,334	38.41	
Annotated in NT	9,721	14.18	
Annotated in KO	5,164	7.53	31
Annotated in SwissProt	20,331	29.65	
Annotated in PFAM	20,197	29.46	
Annotated in GO	21,925	31.98	55
Annotated in KOG	10,268	14.97	26
Annotated in all databases	1,871	2.72	
Annotated in at least one database	29,987	43.74	
Total unigenes	68,547		

### Differential Expression Analysis of Kernels

**FIGURE 1:**
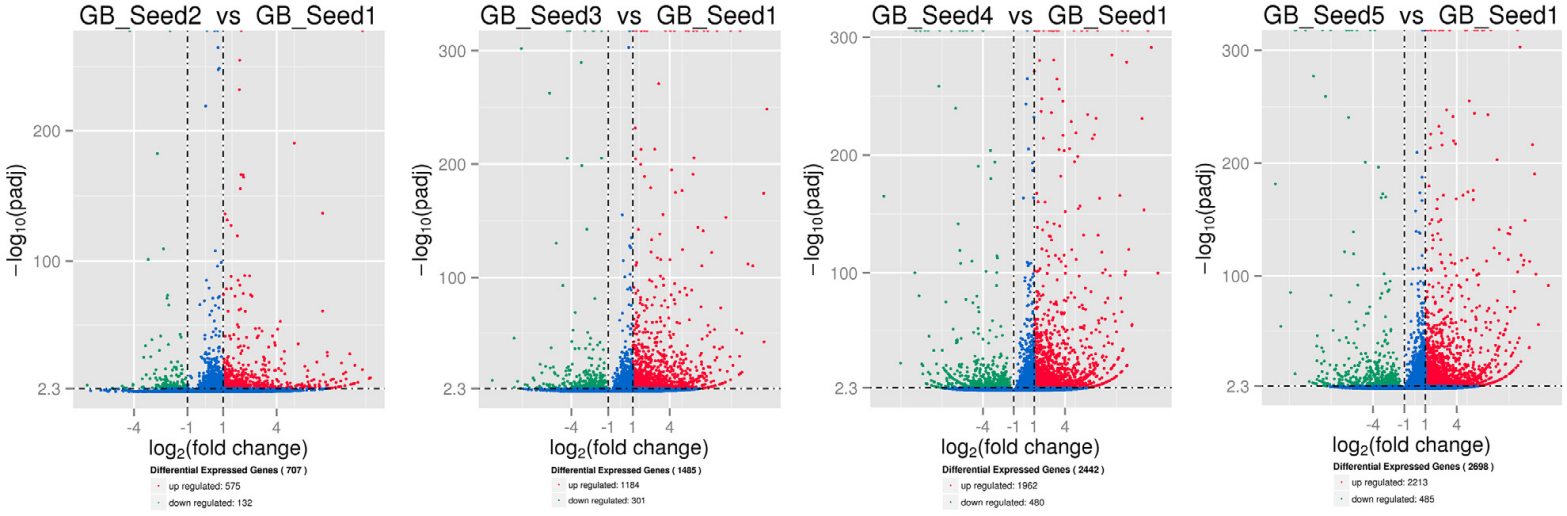
**Differential expressed genesduring kernel developments.** The horizontal axis represents fold changes of gene expression, and the vertical axis represents the statistically significance level. The red dots mean significantly up-regulated genes and the green dots represent significantly down-regulated genes.

**FIGURE 2:**
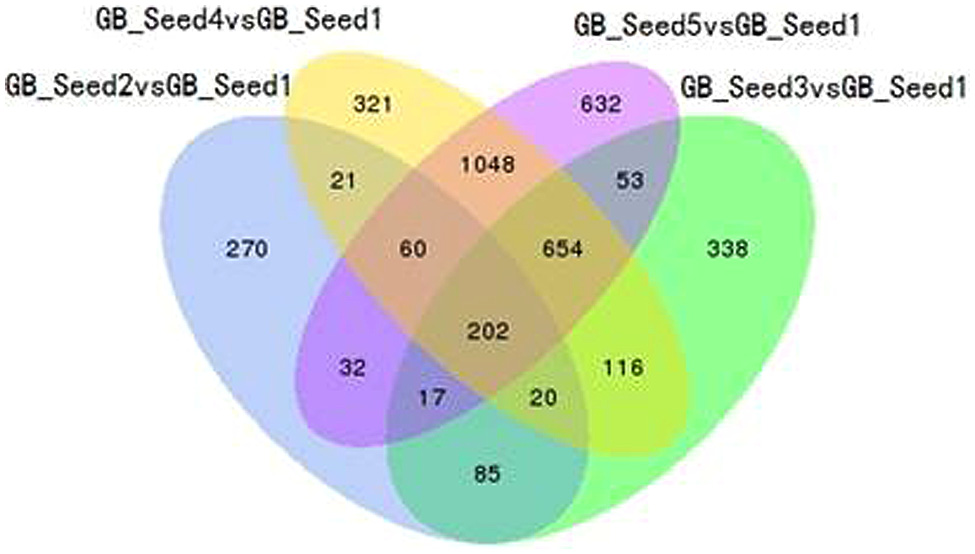
**Venn diagram of differentially expressed genes (DEGs).** All DEGs are clustered into four comparison groups represented by four ellipses. The sum of all the figures in one ellipse represents the number of DEGs in one comparison group (e.g., GB_Seed2 vs. GB_Seed1). The overlapping parts of different ellipses represent the number of DEGs in common from those comparison groups.

The transcriptome assembled by Trinity was employed as the ref, and clean reads of each sample were mapped to this ref. Over 85% of the sequences per sample could be mapped (**Table 4**). In total, 3869 DEGs were identified and the DEG clustering figure using k-means method is contained in the Supplementary File S2. Between GB_Seed2 and GB_Seed1, 707 DEGs were identified, including 575 up-regulated and 132 down-regulated DEGs (**Figure 1**). A total of 1485 DEGs were identified between GB_Seed3 and GB_Seed1, including 1184 up-regulated and 301 down-regulated DEGs. A total of 2442 DEGs were identified between GB_Seed 4 and GB_Seed 1, including 1962 up-regulated and 480 down-regulated DEGs. There were 2698 DEGs between GB_Seed5 and GB_Seed1, 2213 of which were up-regulated and 485 of which were down-regulated. In all four comparison groups (GB_Seed2 vs. GB_Seed1, GB_Seed3 vs. GB_Seed1 and etc.), 202 DEGs in common were identified (**Figure 2**).

**Table 4:** Comparisions between reads and the ref in different samples.

Sample name	Total reads	Total mapped
GB_Seed1	33,453,248	29,019,296 (86.75%)
GB_Seed2	31,775,354	27,172,744 (85.52%)
GB_Seed3	33,397,892	28,679,520 (85.87%)
GB_Seed4	30,896,868	26,442,418 (85.58%)
GB_Seed5	37,692,542	32,282,386 (85.65%)

*Total reads, number of clean reads after sequence filtering. Total mapped, number of clean reads mapped to the ref.*

**FIGURE 3:**
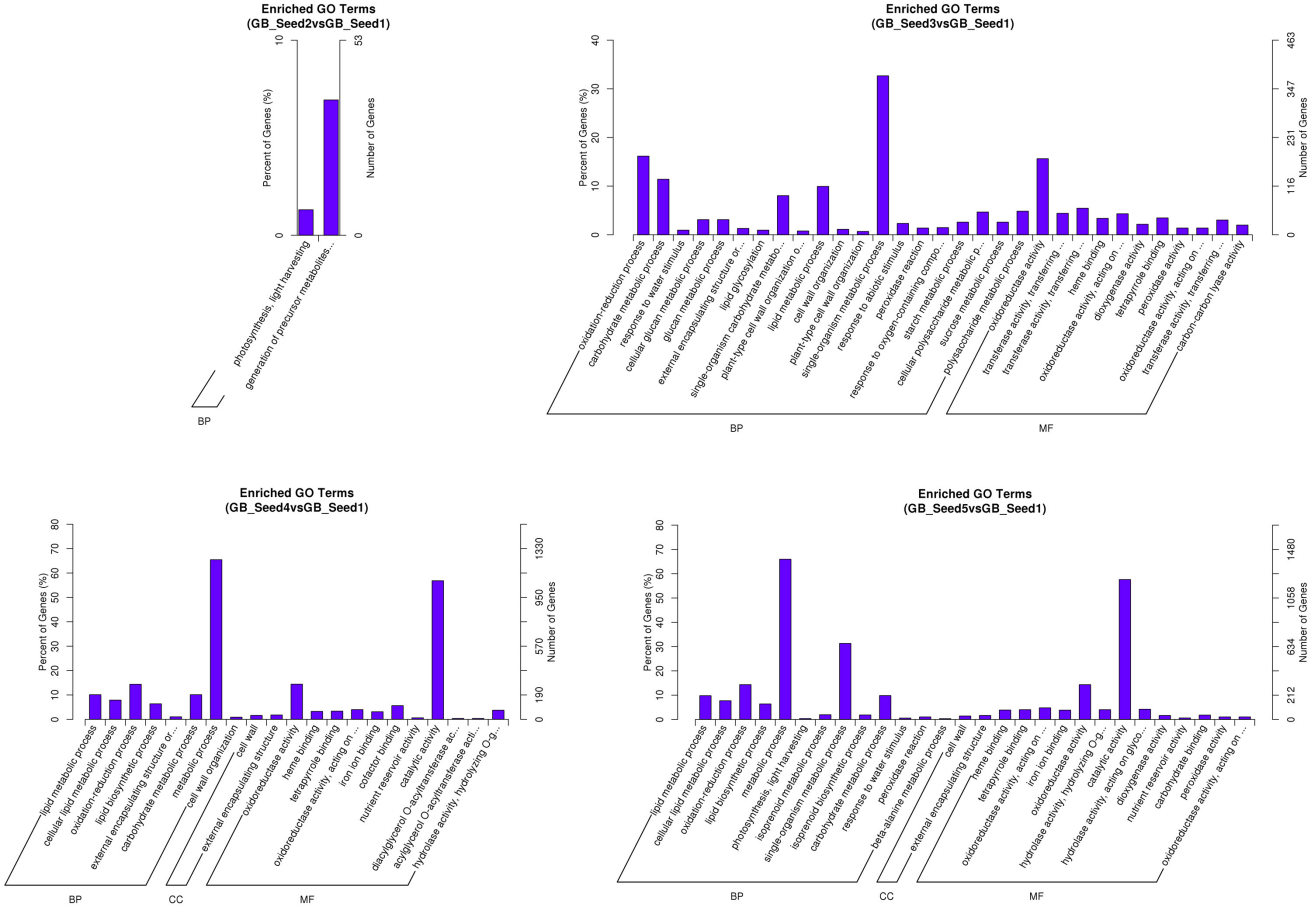
**Functional gene ontology classification of DEGs**.

After screening for DEGs, studying their GO distribution can elucidate the functional differences between different samples. To obtain a better understanding of the DEGs, we not only performed enrichment analysis (GO enrichment and KEGG enrichment) on all DEGs (**Figure 3**), but also divided them into up-regulated and down-regulated groups (Supplemetary File S3). When the corrected *p*-value of one function was less than 0.05, we regarded that function as enriched. The results showed that between GB_Seed2 and GB_Seed1, the genes involved in “photosynthesis, light harvesting” and “generation of precursor metabolites and energy” had the highest degree of enrichment; both of these classifications were down-regulated and belonged to the biological process category. Other significantly down-regulated DEGs included those in the “cytoplasmic part” and “cytoplasm” classifications, belonging to the cellular category. Between GB_Seed3 and GB_Seed1, the DEGs involved in “oxidoreductase activity,” “tetrapyrrole binding,” and “heme binding” were confirmed as up-regulated; all of these classifications belonged to the molecular function category. Other highly enriched DEGs were related to the “single-organism carbohydrate metabolic processes” and “cytoplasmic part.” Between GB_Seed4 and GB_Seed1, the DEGs involved in “oxidoreductase activity,” “lipid metabolic process,” and “cellular lipid metabolic process” were confirmed to be up-regulated, while those involved in “oxidoreductase activity” and “lipid metabolic process” were down-regulated. Other highly enriched genes were related to “metabolic process” and “catalytic activity.” Between GB_Seed5 and GB_Seed1, the major up-regulated DEGs were related to “metabolic process” and “catalytic activity,” while the genes involved in the “oxidation-reduction process” were down-regulated. Other highly enriched DEGs were involved in the “single-organism carbohydrate metabolic process.”

We searched all of the DEGs against the KEGG database to further study the biochemical pathways. KEGG enrichment analysis based on the KEGG pathways was employed to find all pathways that were enriched between the DEGs and annotated genes using a hypergeometric test. The formula for this analysis is shown below:

$$p=1-\sum_{i=0}^{m-1}\frac{\left(\overset{m}{i}\right)\left(\overset{N-M}{n-i}\right)}{\left(\overset{N}{n}\right)}$$

**FIGURE 4:**
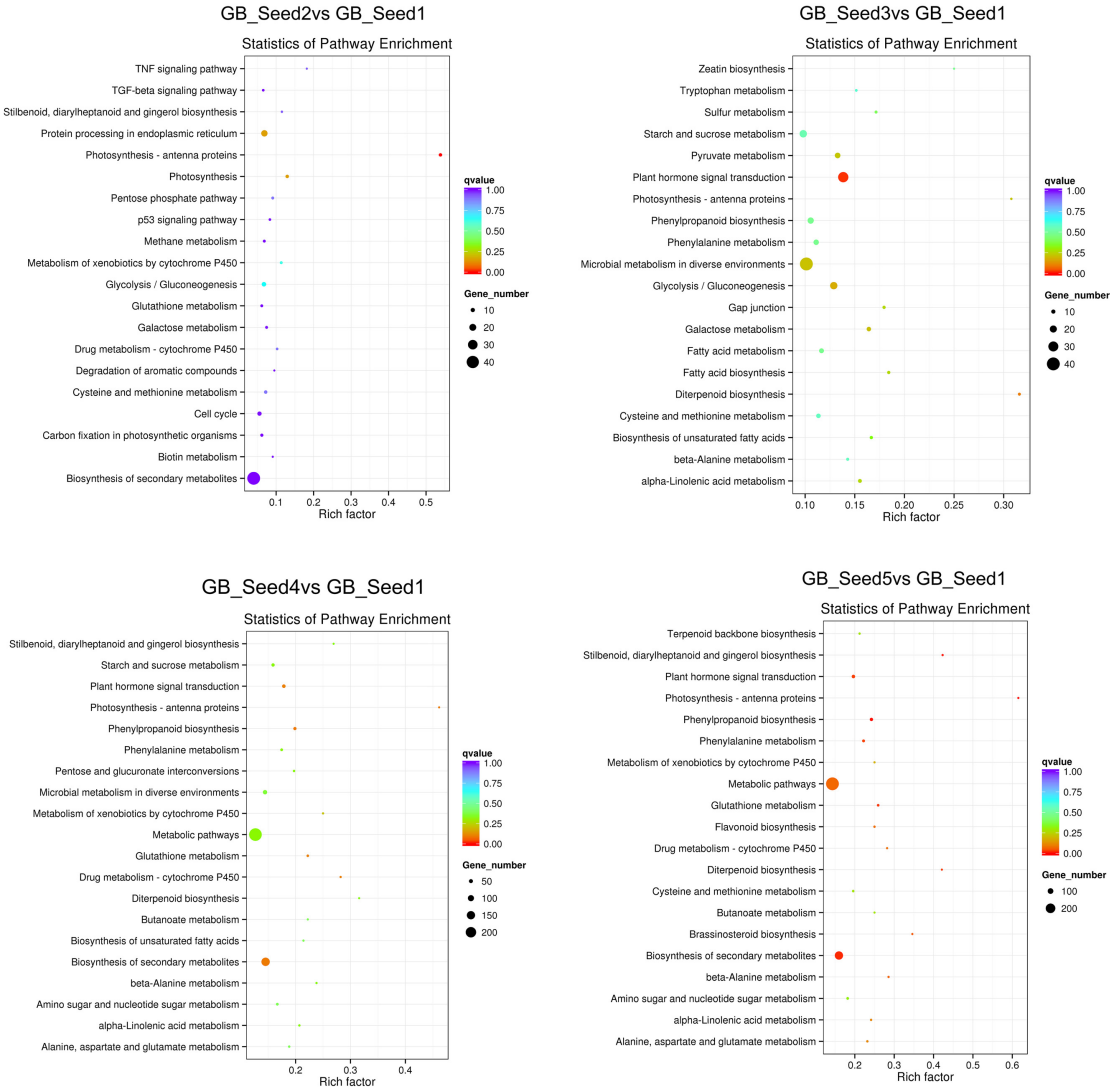
**Kyoto Encyclopedia of Genes and Genomes (KEGG) pathway enrichment of DEGs.** The color is closer to red when the *q*-value is lower.

In this formula, *N* represents the number of genes annotated across all KEGG pathways, and *n* represents the number of DEGs in *N*. *M* represents the number of genes annotated to one specific pathway, and *m* represents the number of DEGs in *M*. We used KOBAS (2.0) to conduct pathway enrichment analysis and set the corrected *p*-value (*q*-value) <0.05. Similarly the process for GO distribution, we searched all DEGs, as well as the up-regulated and down-regulated DEGs separately, against the KEGG. The 20 pathways with the most significant enrichment for each comparison are shown as bar plots, and all pathways are shown if less than 20 were detected. Between GB_Seed2 and GB_Seed1, the most significantly enriched pathway was “photosynthesis-antenna proteins,” which contained down-regulated DEGs. The other possibly enriched pathway in this comparison, “photosynthesis,” was down-regulated as well. Notably, that number of DEGs involved in the “biosynthesis of secondary metabolites” was the largest, although the *q*-value of this pathway was 1. The second largest group of DEGs was “metabolic pathways” (*q*-value = 0.49). Between GB_Seed3 and GB_Seed1, the most significantly enriched pathway was “plant hormone signal transduction,” which was up-regulated together and involved the most DEGs. Other significantly enriched pathways were “photosynthesis-antenna proteins” and “fatty acid biosynthesis,” both of which involved down-regulated DEGs. Between GB_Seed4 and GB_Seed1, although no pathway met the *q*-value requirement, the most DEGs were involved in “metabolic pathways” (*q*-value = 0.22) and the “biosynthesis of secondary metabolites” (*q*-value = 0.09). Both up-regulated and down-regulated DEGs were related to secondary metabolites, and the majority of them were up-regulated in this comparison. Between GB_Seed5 and GB_Seed1, large amounts of genes were differentially expressed (**Figure 4**). The most significantly enriched pathways were “metabolic pathways” and “biosynthesis of secondary metabolites.” Up-regulated DEGs were dominant in the “biosynthesis of secondary metabolites.” Although most DEGs in the “metabolic pathways” were up-regulated, this enrichment was not significant. Other significantly down-regulated DEGs were involved in “photosynthesis-antenna proteins” and “diterpenoid biosynthesis.” Significantly up-regulated DEGs were involved in the “plant hormone signal transduction,” “phenylpropanoid biosynthesis,” and “glutathione metabolism” pathways.

### DEGs in the MEP/MVA Pathways

**FIGURE 5:**
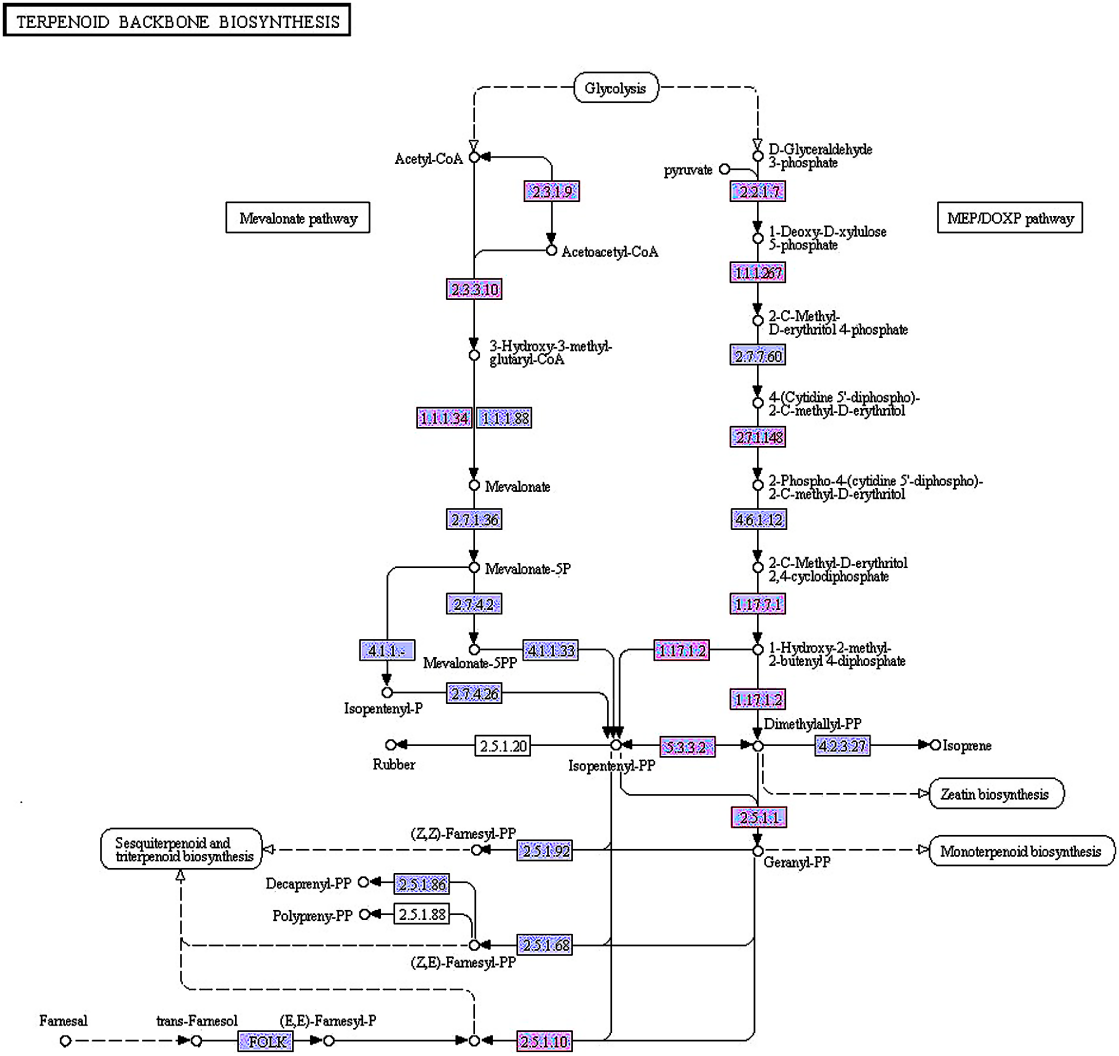
**Differentially expressed genes in the methylerythritol 4-phosphate and mevalonic acid pathways between GB_Seed5 and GB_Seed1.** Red indicates up regulated genes in GB_Seed5 compared with GB_Seed1. The unigene IDs and their log_2_ (fold change) values at different time points are listed below. The higher log_2_ (fold change) value, the more significant level. MEP pathway: 2.2.1.7: comp27284_c0 (0-0-0-4.28); 1.1.1267: comp27939_c0 (0-0-1.32-3.10); 2.7.1.148: comp8253_c0 (0-0-1.63-1.50); 1.17.7.1: comp15978_c0 (0-0-0-1.70); 1.17.7.2: comp37557_c0 (0-0-0-1.51); comp27034_c0 (0-0-1.32-1.51); 5.3.3.2: comp40873_c0 (0-1.54-2.47-2.72); 2.5.1.1: com36057_c0 (0-0-1.79-2.31); comp26079_c0 (0-0-1.47-1.66); MVA pathway: 2.3.1.9: comp27975_c0 (0-0-1.52-2.14); 2.3.3.10: comp38302_c0 (0-0-3.92-3.70); comp38302_c1 (0-0-7.62-6.48); 1.1.1.34: comp38586_c0 (1.54-2.12-6.45-6.15); comp56812_c0 (0-0-3.54-2.18); 2.5.1.10: comp26079_c0 (0-0-1.47-1.66).

A total of 66 unigenes were involved in terpenoid backbone biosynthesis. Comparing to the first time point, a total of 12 unigenes were differentially expressed in the fifth period. Since the number of DEGs between GB_Seed5 and GB_Seed1 was the largest, we paid more attention to the KEGG pathway annotations between these two time points (**Figure 5**). Between GB_Seed4 and GB_Seed1, 11 unigenes were differentially expressed. Most of the DEGs were similar with that between GB_Seed5 and GB_Seed1 and the fold changes in this comparison were relatively lower. Between GB_Seed3 and GB_Seed1, two unigenes (comp38586_c0 and comp40873_c0) were identified up-regulated and between GB_Seed2 and GB_Seed1, one unigene (comp38586_c0) was identified up-regulated.

In the MEP pathway between GB_Seed5 and GB_Seed1, comp27284_c0, annotated as 1-deoxy-D-xylulose-5-phosphate synthase (*DXS*), was up-regulated. Comp27939_c0, annotated as 1-deoxy-D-xylulose-5-phosphate reductoisomerase (*DXR*), was up-regulated. *DXR* is the key enzyme that catalyzes 1-deoxy-D-xylulose 5-phosphate into 2-*C*-methyl-D-erythritol 4-phosphate (Takahashi et al., 1998). The up-regulated gene codes for 4-diphosphocytidyl-2-*C*-methyl-D-erythritol kinase (*ispE*), which catalyzes the conversion of 4-(cytidine 5’-diphospho)-2-*C*-methyl-D-erythritol into 2-phospho-4-(cytidine 5’-diphospho)-2-*C*-methyl-D-erythritol(comp8253_c0). The gene coding for (E)-4-hydroxy-3-methylbut-2-enyl-diphosphate synthase, which catalyzes the conversion of 2-*C*-methyl-D-erythritol 2, 4-cyclodiphosphate into 1-hydroxy-2-methyl-2-butenyl 4-diphosphate for further reactions that generate DMAPP and IPP, was up-regulated as well. The up-regulated gene encoded1-hydroxy-2-methyl-2-butenyl 4-diphosphate reductase, which catalyzing the conversion of 1-hydroxy-2-methyl-2-butenyl 4-diphosphate into IPP and DMAPP (Rohdich et al., 2002) and two related transcripts were identified (comp37557_c0, comp27034_c0). Two homologous transcripts in the constructed library, comp36057_c0 and comp26079_c0, were annotated as FPP synthase (*GPS*) that catalyze the conversion of DMAPP into geranyl diphosphate (Cunillera et al., 1996) and transform IPP into FPP, respectively. Comp40873_c0, annotated as *IDI*, was up-regulated in this comparison.

In the MVA pathway between GB_Seed5 and GB_Seed1, the gene encoding acetyl-CoA *C*-acetyltransferase was also up-regulated. This gene has not been cloned in *G. biloba*, and we annotated comp27975_c0 as this enzyme. Comp38302_c1 and comp38302_c2, annotated as hydroxymethylglutaryl-CoA synthases that convert acetyl-CoA into HMG-CoA, were also up-regulated. The gene coding for farnesol dehydrogenase (*FLDH*) which oxidizes (2E, 6E)-farnesol to 2-trans, 6-trans-farnesal (Bhandari et al., 2010), was up-regulated as well (comp11165_c0).

### Verification by Real-time Quantitative PCR

**FIGURE 6:**
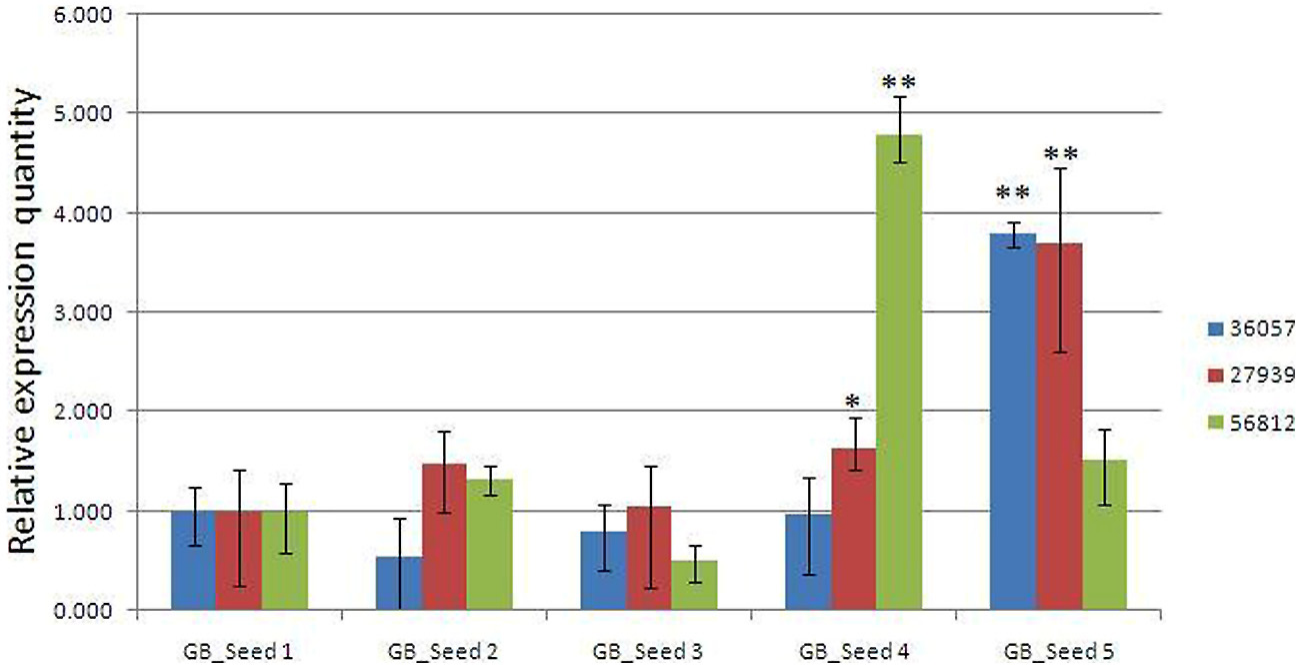
**Real-time PCR validations of 3 genes involved in MEP and MVA pathways.** Comp36057_c0: *GPS*; comp27939_c0: *DXR*; comp56812_c0: *HMGR*. The vertical bars represented the stardard error. ^∗^*p* < 0.05, ^∗∗^*p* < 0.01.

To confirm the reliability of the transcriptome data, the transcriptome level of 3 unigenes were examined by real-time quantitative PCR (**Figure 6**). The three unigenes showed expression patterns which were consistent with the transcriptome data.

## Discussion

Over the past few years, next-generation sequencing (NGS) technology has brought profound changes to genomics and genetics, and sequencing rates have become continually faster while costs have continually decreased (Mardis, 2008). Among representative NGS platforms, the Illumina HiSeq 2000 platform is widely used for deep sequencing due to its high throughput and favorable cost performance. Expressed sequence tag (EST) sequencing is a useful method to understand molecular mechanisms underlying physiological and morphological traits. Sequencing technology was first developed in 1977 but was problematic due to its high labor and cost requirements. After years of improvement, NGS technology methods have improved the speeds, convenience, and cost of sequencing. Because the Illumina Hiseq2000 platform has the best cost performance among NGS methods, it has been widely used for the deep sequencing of model and non-model organisms (De Donato et al., 2013). In this study, over 185 million raw reads and 167 million clean reads, representing 25.08 Gb, were obtained using a HiSeq2000 machine. After filtering, the high-quality sequences were assembled using Trinity, with a minimum length cutoff of 200 bp. A total of 112,946 singletons and 68,547 unigenes were generated. The average length of these unigenes was 870 bp; the shortest was 201 bp, and the longest was 18,087 bp.

Based on this data, we conducted the first sequencing of *G. biloba* kernels at different time points, and we attempted to reveal the biosynthetic mechanisms of ginkgolide and bilobalide in these kernels. These data will contribute to the preservation of *Ginkgo* germplasm resources and further functional genomics analyses in this species. A total of 3869 unigenes were identified, most of which were annotated by the GO and KEGG databases. As we manually divided the seeds into five time points, the DEGs at different stages provide effective references for understanding the details of gene expression and regulation over the growth of *G. biloba* seeds.

A previous 454 GX FLX sequencing study of *Gingko biloba* leaves in Lin et al. (2011) generated a total of 64,057 ESTs and 22,304 unigenes, with an average length of 402 bp. Nine putative transcripts involved in MEP pathway and four putative transcripts involved in MVA pathway were identified in that study and in our research, these related unigenes were identified as well. Since most previous studies focused on *G. biloba* leaves together with the putative transcripts identification, the kernels transcriptome was sequenced for the first time and the results would benefit further study of functional genomics and developmental biology. By Illumina sequencing in our study, a total of 29,987 (43.74%) unigenes were annotated in at least one database, while 20,331 unigenes were unknown. Of these unigenes, 5164 were annotated in the KO database, covering 31 categories. In Lin and his colleagues’ work, 64.5% of the unigenes were annotated and the percentage was higher than that in our research. These un-annotated unigenes may represent more unique functional genes in kernels. The discrepancy about unigene annotation would result from the biological tissue differences. Besides, much more transcripts were obtained in our study than that of Lin and his colleagues’, and more rare transcripts would be sequenced.

We mainly focused on the biosynthesis of the common precursor GGPP, considering the complex mechanisms of diterpenoid and sesquiterpenoid synthesis. Comparing to the first time point, no unigenes concerning in the MEP and MVA pathways was significantly down-regulated. It is possible that ginkgolide and bilobalide were both generated rapidly during the seed maturation process. Differential expression analysis revealed that many genes involved in the MEP pathway toward GGPP were up-regulated, such as 1-deoxy-D-xylulose-5-phosphate reductoisomerase, 4-diphosphocytidyl-2-*C*-methyl-D-erythritol kinase, and 1-hydroxy-2-methyl-2-butenyl 4-diphosphate and FPP synthases. Most of these genes were expressed at the fourth and the fifth time points, while fewer expressed genes involved in the MVA pathway were identified.

According to the qRT-PCR results, two unigenes (comp36057_c0 and comp_27939c0), encoding GPS and DXR enzymes respectively, were both enzymes in the MEP pathway. These two unigenes maintained relatively stable levels at first, and then they were up-regulated significantly at the last two periods and finally reached the peak at the fifth time point. One another unigene (comp56812_c0) encoded HMGR, the enzyme involved in the MVA pathway. The expression levels reached a peak at the fourth period and then suddenly dropped to a much lower level at the last time point, although still higher than that at the first time point. Is it possible that ginkgolide biosynthesis would occur earlier than bilobalide synthesis in *Ginkgo* kernels? Would it be supposed that two synthesis pathways compete for the utilization of their common precursors? The validation to these hypothesizes requires much following work, and some key genes involved in the two pathways are being functionally analyzed in our laboratory, providing a foundation for the further study of ginkgolide and bilobalide biosynthesis in *Gingko* kernels.

## Conflict of Interest Statement

The authors declare that the research was conducted in the absence of any commercial or financial relationships that could be construed as a potential conflict of interest.

## References

[B1] Anders S. Huber W. (2010). Differential expression analysis for sequence count data. *Genome Biol.* 11 R106 10.1186/gb-2010-11-10-r106 PMC3218662 20979621

[B2] Bhandari J. Fitzpatrick A. H. Crowell D. N. (2010). Identification of a novel abscisic acid-regulated farnesol dehydrogenase from *Arabidopsis*. *Plant Physiol.* 154 1116–1127. 10.1104/pp.110.157784 20807998 PMC2971593

[B3] Cunillera N. Arró M. Delourme D. Karst F. Boronat A. Ferrer A. (1996). *Arabidopsis thaliana* contains two differentially expressed farnesyl-diphosphate synthase genes. *J. Biol. Chem.* 271 7774–7780. 10.1074/jbc.271.13.7774 8631820

[B4] De Donato M. Peters S. O. Mitchell S. E. Hussain T. Imumorin I. G. (2013). Genotyping-by-sequencing (GBS): a novel, efficient and cost-effective genotyping method for cattle using next-generation sequencing. *PLoS ONE* 8:e62137 10.1371/journal.pone.0062137 PMC3656875 23690931

[B5] Gao S. Lin J. Liu X. Deng Z. Li Y. Sun X. (2006). Molecular cloning, characterization and functional analysis of a 2C-methyl- D-erythritol 2, 4-cyclodiphosphate synthase gene from *Ginkgo biloba*. *J. Biochem. Mol. Biol.* 39 502–510. 10.5483/BMBRep.2006.39.5.502 17002869

[B6] Gong W. (2008). Phylogeography of a living fossil: pleistocene glaciations forced *Ginkgo biloba* L. (Ginkgoaceae) into two refuge areas in China with limited subsequent postglacial expansion. *Mol. Phylogenet. Evol.* 48 1094–1105. 10.1016/j.ympev.2008.05.003 18554931

[B7] Götz S. García-Gómez J. M. Terol J. Williams T. D. Nagaraj S. H. Nueda M. J. (2008). High-throughput functional annotation and data mining with the Blast2GO suite. *Nucleic Acids Res.* 36 3420–3435. 10.1093/nar/gkn176 18445632 PMC2425479

[B8] Grabherr M. G. Haas B. J. Yassour M. Levin J. Z. Thompson D. A. Amit I. (2011). Full-length transcriptome assembly from RNA-Seq data without a reference genome. *Nat. Biotechnol.* 29 644–652. 10.1038/nbt.1883 21572440 PMC3571712

[B9] Horáková L. Licht A. Sandig G. Jakstadt M. Duracková Z. Grune T. (2003). Standardized extracts of flavonoids increase the viability of PC12 cells treated with hydrogen peroxide: effects on oxidative injury. *Arch. Toxicol.* 77 22–29. 10.1007/s00204-002-0409-8 12491037

[B10] Kim J. H. Lee K. I Chang Y. J. Kim S. U. (2012). Developmental pattern of *Ginkgo biloba* levopimaradiene synthase (GbLPS) as probed by promoter analysis in *Arabidopsis thaliana*. *Plant Cell Rep.* 31 1119–1127. 10.1007/s00299-012-1232-1 22311479

[B11] Kim S.-M. Kuzuyama T. Chang Y.-J. Kwon H.-J. Kim S.-U. (2006a). Cloning and functional characterization of 2-C-methyl-D-erythritol 4-phosphate cytidyltransferase (GbMECT) gene from *Ginkgo biloba*. *Phytochemistry* 67 1435–1441. 10.1016/j.phytochem.2006.05.034 16828818

[B12] Kim S. M. Kuzuyama T. Chang Y. J. Song K. S. Kim S. U. (2006b). Identification of class 2 1-deoxy-D-xylulose 5-phosphate synthase and 1-deoxy-D-xylulose 5-phosphate reductoisomerase genes from *Ginkgo biloba* and their transcription in embryo culture with respect to ginkgolide biosynthesis. *Planta Med.* 72 234–240. 10.1055/s-2005-916180 16534728

[B13] Kressmann S. Müller W. Blume H. (2002). Pharmaceutical quality of different *Ginkgo biloba* brands. *J. Pharm. Pharmacol.* 54 661–669. 10.1211/0022357021778970 12005361

[B14] Li B. Dewey C. N. (2011). RSEM: accurate transcript quantification from RNA-Seq data with or without a reference genome. *BMC Bioinformatics* 12:323 10.1186/1471-2105-12-323 PMC3163565 21816040

[B15] Lin C. Wu C.-S. Huang Y.-Y. Chaw S.-M. (2012). The complete chloroplast genome of *Ginkgo biloba* reveals the mechanism of inverted repeat contraction. *Genome Biol. Evol.* 4 347–381. 10.1093/gbe/evs021 PMC3318433 22403032

[B16] Lin S.-J. Yang T.-H. Chen Y.-H. Chen J.-W. Kwok C.-F. Shiao M.-S. (2002). Effects of *Ginkgo biloba* extract on the proliferation of vascular smooth muscle cells in vitro and on intimal thickening and interleukin-1β expression after balloon injury in cholesterol-fed rabbits in vivo. *J. Cell. Biochem.* 85 572–582. 10.1002/jcb.10151 11967997

[B17] Lin X. Zhang J. Li Y. Luo H. Wu Q. Sun C. (2011). Functional genomics of a living fossil tree, Ginkgo, based on next-generation sequencing technology. *Physiol. Plant.* 143 207–218. 10.1111/j.1399-3054.2011.01500.x 21834857

[B18] Mao X. Cai T. Olyarchuk J. G. Wei L. (2005). Automated genome annotation and pathway identification using the KEGG Orthology (KO) as a controlled vocabulary. *Bioinformatics* 21 3787–3793. 10.1093/bioinformatics/bti430 15817693

[B19] Mardis E. R. (2008). Next-generation DNA sequencing methods. *Annu. Rev. Genomics Hum. Genet.* 9 387–402. 10.1146/annurev.genom.9.081307.164359 18576944

[B20] Nakanishi K. (1967). The ginkgolides. *Pure Appl. Chem.* 14 89–113. 10.1351/pac196714010089 6036635

[B21] Oken B. S. Storzbach D. M. Kaye J. A. (1998). The efficacy of *Ginkgo biloba* on cognitive function in Alzheimer disease. *Arch. Neurol.* 55 1409–1415. 10.1001/archneur.55.11.1409 9823823

[B22] Rodríguez M. Ringstad L. Schäfer P. Just S. Hofer H. Malmsten M. (2007). Reduction of atherosclerotic nanoplaque formation and size by < i > *Ginkgo biloba*(EGb 761) in cardiovascular high-risk patients. *Atherosclerosis* 192 438–444. 10.1016/j.atherosclerosis.2007.02.021 17397850

[B23] Rohdich F. Hecht S. Gärtner K. Adam P. Krieger C. Amslinger S. (2002). Studies on the nonmevalonate terpene biosynthetic pathway: metabolic role of IspH (LytB) protein. *Proc. Natl. Acad. Sci. U.S.A.* 99 1158–1163. 10.1073/pnas.032658999 11818558 PMC122160

[B24] Rohmer M. (1999). The discovery of a mevalonate-independent pathway for isoprenoid biosynthesis in bacteria, algae and higher plants†. *Nat. Prod. Rep.* 16 565–574. 10.1039/a709175c 10584331

[B25] Sabater-Jara A. B. Souliman-Youssef S. Novo-Uzal E. Almagro L. Belchí-Navarro S. Pedreño M. A. (2013). Biotechnological approaches to enhance the biosynthesis of ginkgolides and bilobalide in *Ginkgo biloba*. *Phytochem. Rev.* 12 191–205. 10.1007/s11101-013-9275-7

[B26] Sasaki Y. Noguchi T. Yamamoto E. Giddings J. C. Ikeda K. Yamori Y. (2002). Effects of *Ginkgo biloba* extract (EGb 761) on cerebral thrombosis and blood pressure in stroke-prone spontaneously hypertensive rats. *Clin. Exp. Pharmacol. Physiol.* 29 963–967. 10.1046/j.1440-1681.2002.03761.x 12366386

[B27] Shen G. Pang Y. Wu W. Liao Z. Zhao L. Sun X. (2006). Cloning and characterization of a root-specific expressing gene encoding 3-hydroxy-3-methylglutaryl coenzyme A reductase from *Ginkgo biloba*. *Mol. Biol. Rep.* 33 117–127. 10.1007/s11033-006-0014-7 16817021

[B28] Storey J. D. Tibshirani R. (2003). Statistical significance for genomewide studies. *Proc. Natl. Acad. Sci. U.S.A.* 100 9440–9445. 10.1073/pnas.1530509100 12883005 PMC170937

[B36] Takahashi S. Kuzuyama T. Watanabe H. Seto H. (1998). A 1-deoxy-D-xylulose 5-phosphate reductoisomerase catalyzing the formation of 2-*C*-methyl-D-erythritol 4-phosphate in an alternative nonmevalonate pathway for terpenoid biosynthesis. *Proc. Natl. Acad. Sci. U.S.A.* 95 9879–9884. 10.1073/pnas.95.17.9879 9707569 PMC21430

[B29] Trapnell C. Williams B. A. Pertea G. Mortazavi A. Kwan G. van Baren M. J. (2010). Transcript assembly and quantification by RNA-Seq reveals unannotated transcripts and isoform switching during cell differentiation. *Nat. Biotechnol.* 28 511–515. 10.1038/nbt.1621 20436464 PMC3146043

[B30] van Beek T. A. (2005). Ginkgolides and bilobalide: their physical, chromatographic and spectroscopic properties. *Bioorg. Med. Chem.* 13 5001–5012. 10.1016/j.bmc.2005.05.056 15993092

[B31] Wang Y.-Q. Shen J.-K. Berglund T. Ohlsson A. B. Tang X.-F. Zhou Z.-K. (2010). Analysis of expressed sequence tags from Ginkgo mature foliage in China. *Tree Genet. Genomes* 6 357–365. 10.1007/s11295-009-0254-5

[B32] Wu C. S. Chaw S. M. Huang Y. Y. (2013). Chloroplast phylogenomics indicates that *Ginkgo biloba* is sister to cycads. *Genome Biol. Evol.* 5 243–254. 10.1093/gbe/evt001 23315384 PMC3595029

[B33] Yilmaz H. H. Gormez O. Hastar E. Yildirim D. Aksoy M. C. (2010). Garlic burn in a patient with trigeminal neuralgia: a case report. *Eur. J. Dent.* 4 88–90. 20046486 PMC2798796

[B34] Young M. D. Wakefield M. J. Smyth G. K. Oshlack A. (2010). Method Gene ontology analysis for RNA-seq: accounting for selection bias. *Genome Biol.* 11 R14 10.1186/gb-2010-11-2-r14 PMC2872874 20132535

[B35] Zhou Z. Zheng S. (2003). Palaeobiology: the missing link in Ginkgo evolution. *Nature* 423 821–822. 10.1038/423821a 12815417

