# Supplementary material for: Transcriptome analysis of Ginkgo biloba kernels
Source: Front Plant Sci. 2015 Oct 6;6:819. doi: 10.3389/fpls.2015.00819 (PMC4593864; doi:10.3389/fpls.2015.00819)
Supplement: Supplementary file 1 [file Data_Sheet_1.DOCX]

**Supplementary Documents file**

**Supplementary File 1: Functional annotation and classification**


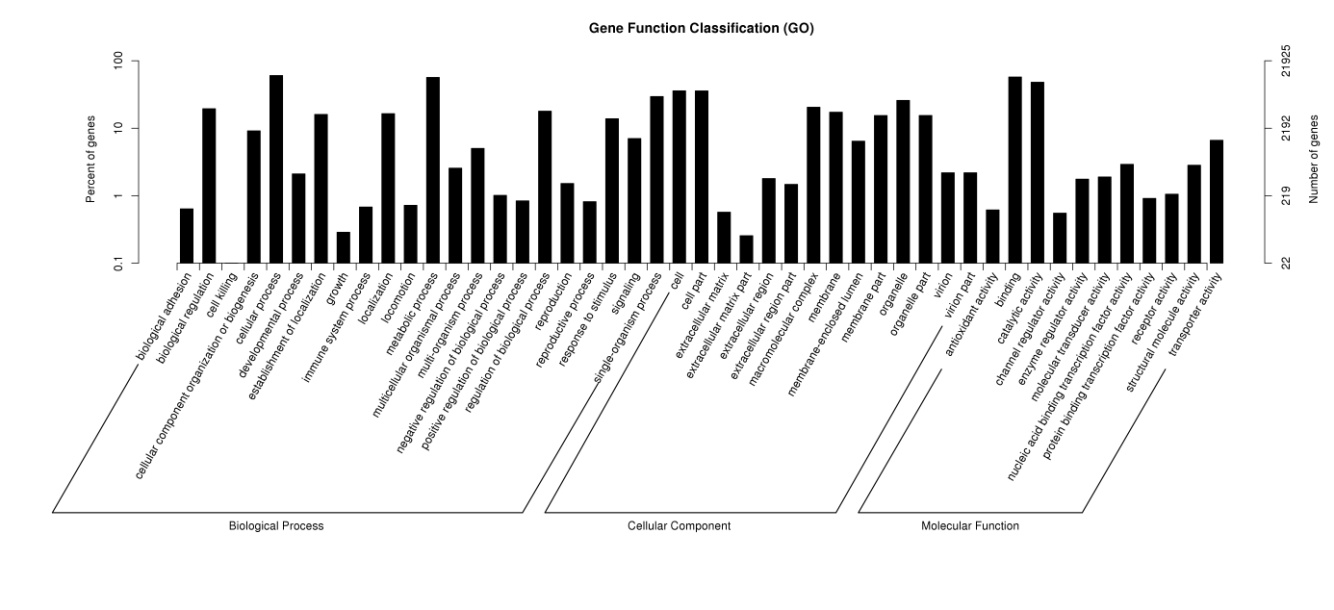


**Gene ontology classification of the assembled unigenes**


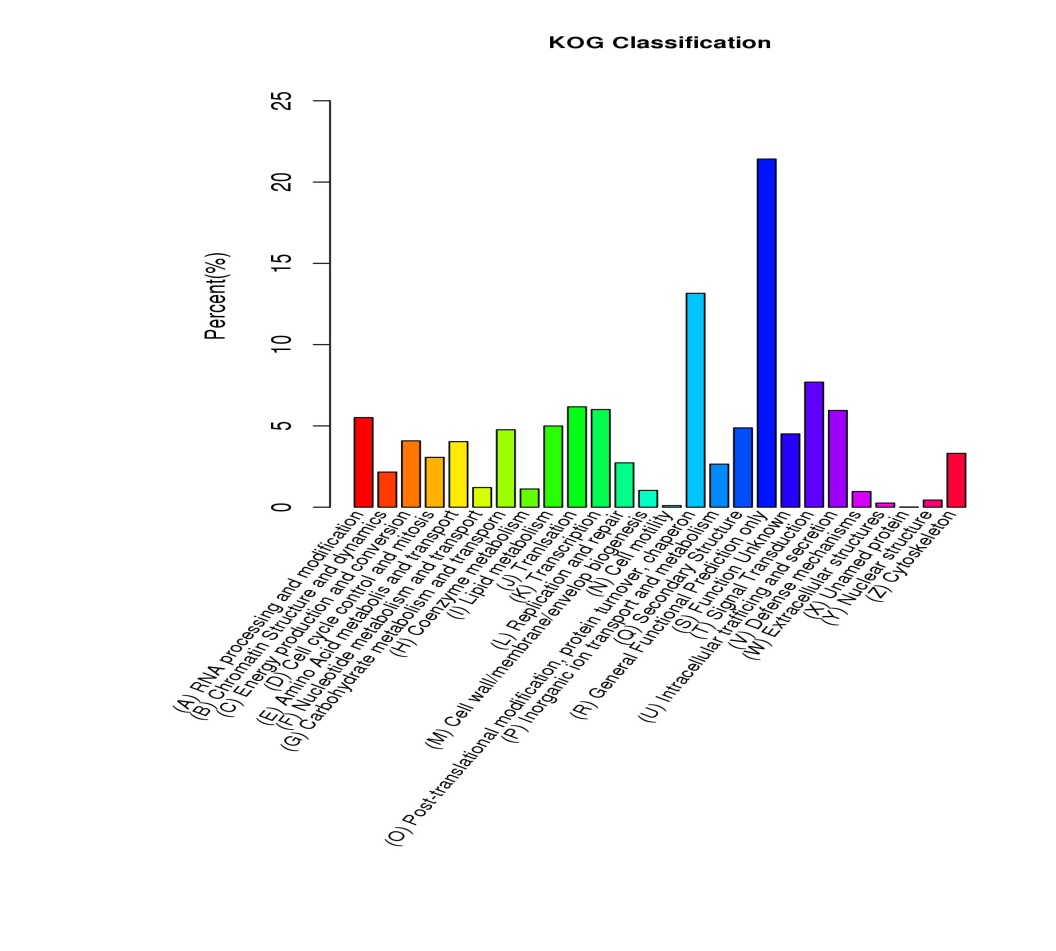


**KOG functional classification of the assembled unigenes**


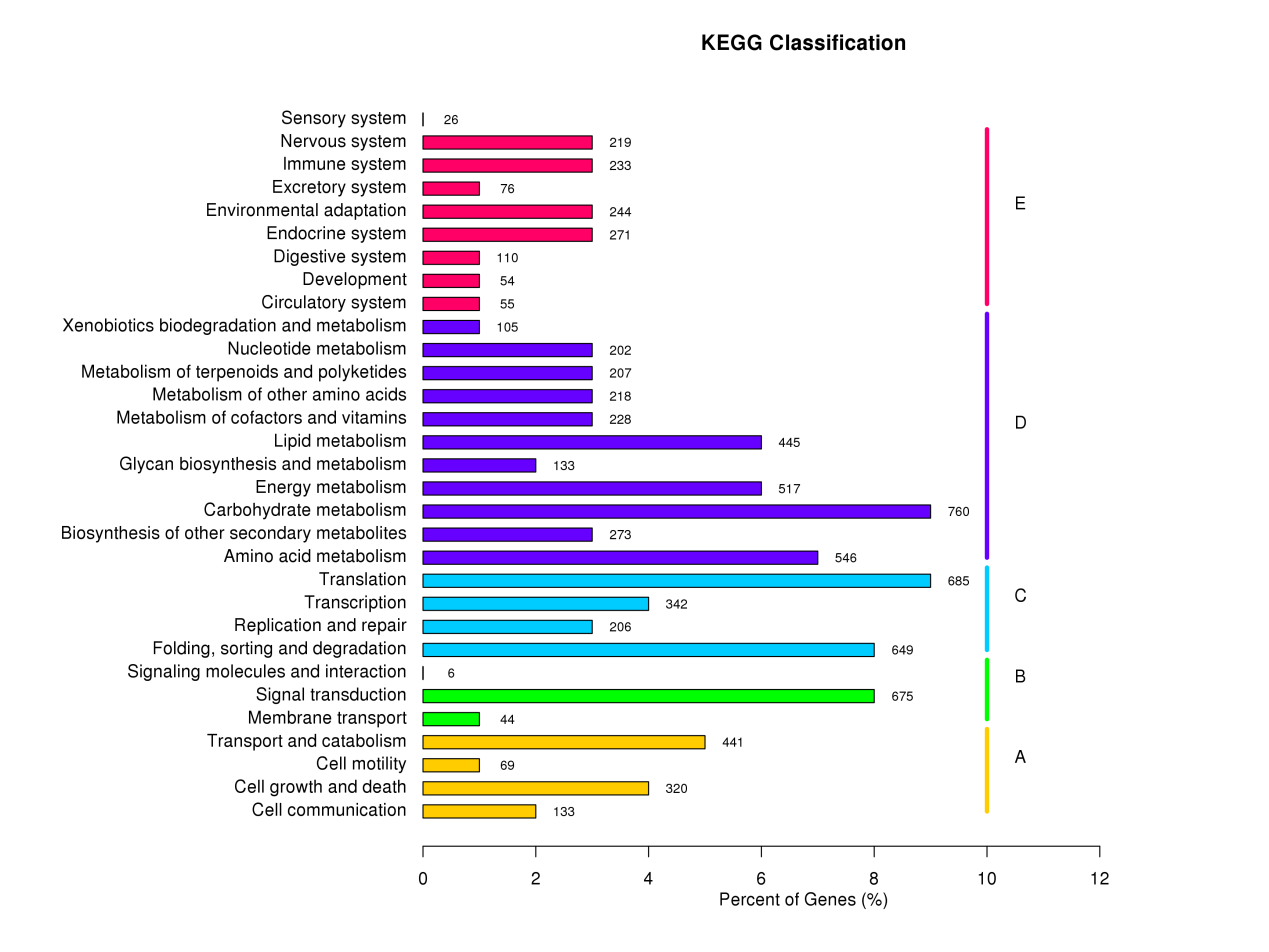


**KEGG classification of the assembled unigenes (**A: Cellular Processes; B: Environmental Information Processing; C: Genetic Information Processing; D: Metabolism; E: Organismal Systems)

**Supplementary File 2:**

**
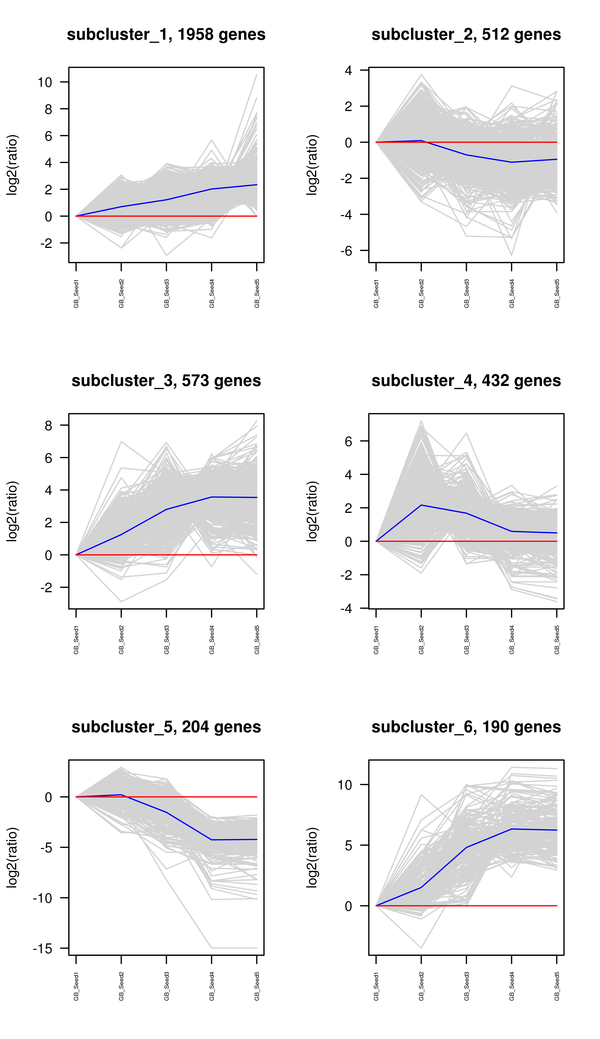
**

**Dendrogram of DEGs with k-means method**

The grey lines represent the relative expression quantity of unigenes at different time points, and the blue lines are the average expression quantity of all the unigenes in each subcluster.

**Supplementary File 3:**

**
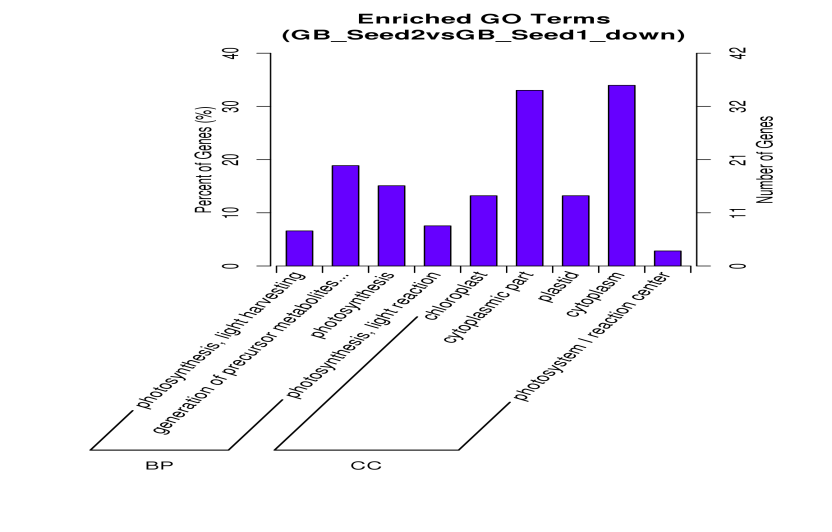
**

**
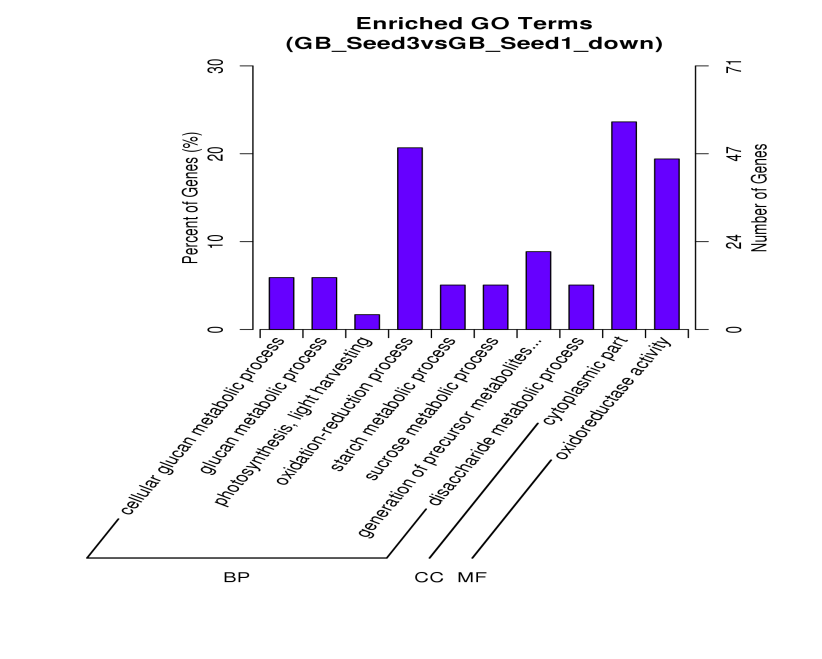

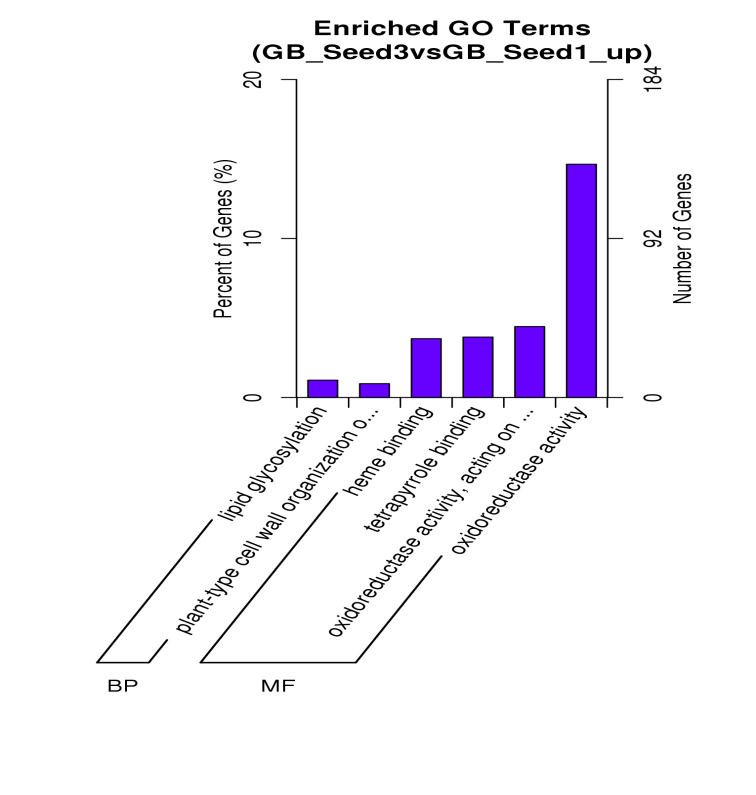
**

**
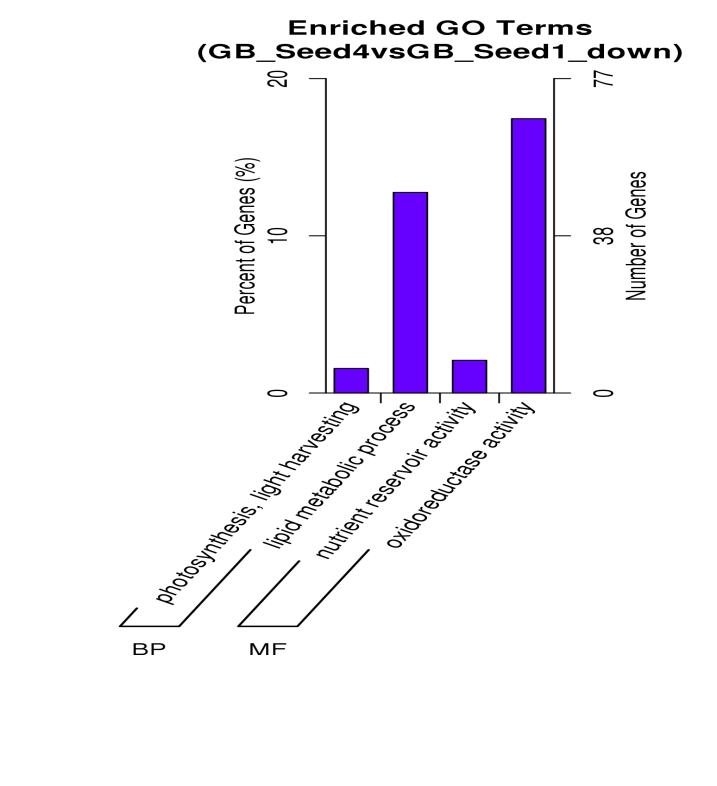
**

**
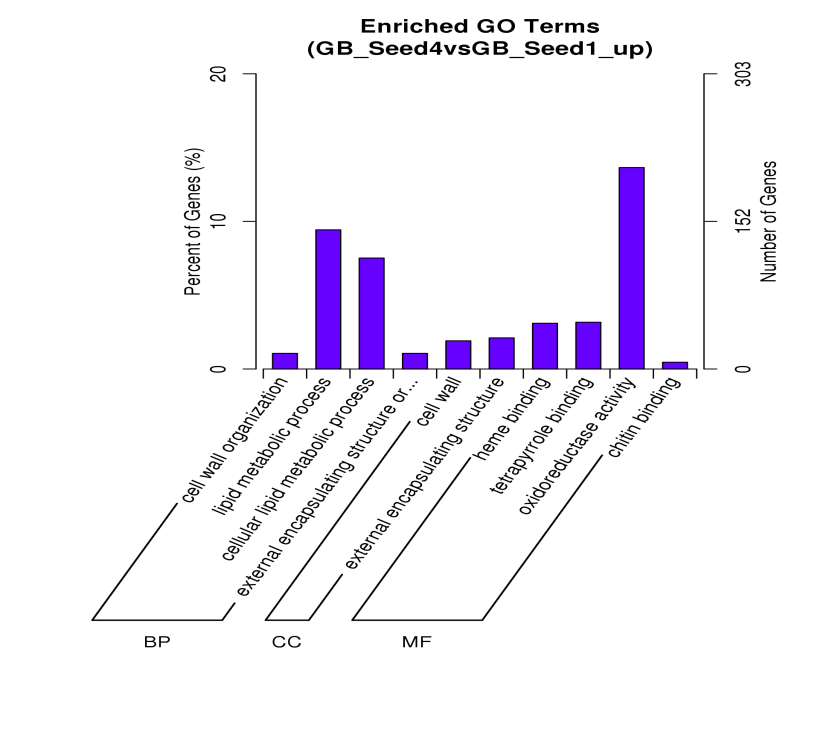
**

**
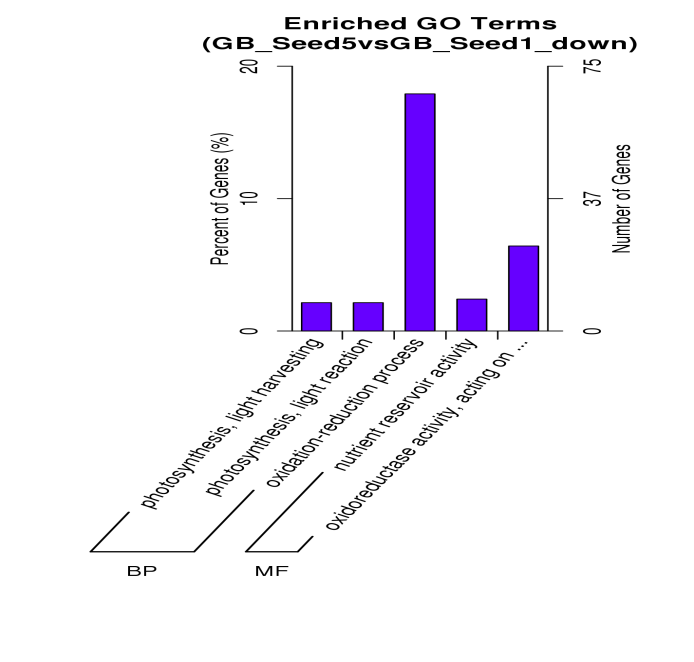
**

**
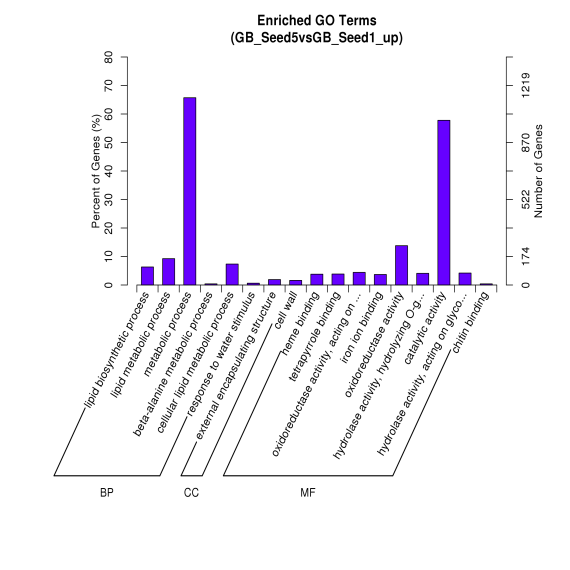
**

**GO classification of up-regulated and down-regulated DEGs**
